# Optical Coherence Tomography Angiography Compared with Indocyanine Green Angiography in Central Serous Chorioretinopathy

**DOI:** 10.1038/s41598-019-42623-x

**Published:** 2019-04-16

**Authors:** Jie Hu, Jinfeng Qu, Zhenyu Piao, Yuou Yao, Guosheng Sun, Mengyang Li, Mingwei Zhao

**Affiliations:** Ophthalmology Optometry Centre, Peking University People’s Hospital, Beijing Key Laboratory of Diagnosis and Therapy of Retinal and Choroid Diseases, Beijing, 100044 China

## Abstract

The purpose of this article is to compare optical coherence tomography angiography (OCTA) and indocyanine green angiography (ICGA) in patients with central serous chorioretinopathy (CSC). OCTA, ICGA and fluorescein angiography (FA) images of all enrolled patients were collected and compared. Abnormal areas were annotated on en face choriocapillaris OCTA and ICGA images and compared with each other. We found three main types of anomalies in choriocapillaris OCTA images: type A, coarse granulated high reflective area (61 eyes [92.4%]); type B, roundish dark halo around Type A (54 eyes [81.8%]); and type C, coarse granulated low reflective area (66 eyes [100%]). There were 54 eyes (81.8%) that exhibited all three types abnormalities, 7 (10.6%) had only type A and C abnormalities, and 5 (7.6%) had only type C abnormalities. The Mean JI of type A on OCTA and hyperfluorescence area on ICGA was 0.84 ± 0.15 and 0.82 ± 0.23 for grader 1 and 2, respectively. Type A area on OCTA had a statistically larger area than hyperfluorescence on ICGA (P = 0.01 [paired t-test]). In summary, abnormalities were found on OCTA images of CSC. Coarse granulated high reflective area in OCTA corresponded well with the hyper-permeability area in ICGA in most of the eyes.

## Introduction

Central serous chorioretinopathy (CSC) is a disorder characterized by serous retinal detachment with or without retinal pigment epithelial (RPE) detachment, and associated with hyper-permeability of the choriocapillary layer. A higher prevalence of CSC among men in their fourth and fifth decades of life has been observed, with the male: female ratio ranging from 2.7:1 to 7:1^1^. CSC incidence varies among ethnic groups, and a higher frequency has been suspected among Asians, Caucasians, and Hispanics compared with African Americans^1^. CSC patients report symptoms including blurred vision, relative central scotoma, metamorphopsia, moderate dyschromatopsia, hypermetropization, and micropsia, and reduced contrast sensitivity^2^. Bilateral, multifocal cases of CSC, with worsened visual acuity, have been reported more frequently in Asians^1–3^. In most cases, acute CSC (disease duration ≤6 months) is self-limiting with favorable outcome; however, 30% to 50%^4^ of acute CSC may be associated with permanent vision loss or recur within one year. Chronic CSC (disease duration >6 months) is characterized by diffuse leakage on fluorescein angiography (FA), widespread RPE alterations and photoreceptor defects, resulting in progressive and inevitable vision loss^5^. Despite observations, CSC with paramacular leakage points can be treated with laser photocoagulation. For individuals with CSC and leakage points directly on the macula, half-dose photodynamic therapy (HD-PDT) is the mainstay treatment choice^6^.

In addition to a full clinical examination, FA, indocyanine green angiography (ICGA), and optical coherence tomography (OCT) can help diagnose CSC^7^. OCT reveals subretinal fluid (SRF), with or without serous pigment epithelial detachment (PED), and the thickening of choroid. Intraretinal fluid (IRF) and macular cysts may be encountered in some patients with chronic CSC. A double-layer sign (DLS), defined as an undulated RPE layer and intact underlying Bruch’s membrane, appears in 87% of chronic and 63% of acute CSC patients^8^. FA reveals one or multiple leakage points and/or diffuse RPE alterations. ICGA reveals choriocapillary hyperpermeability areas with hyperfluorescence. ICGA often reveals a more diffuse hyperfluorescent area compared with FA. In some cases, ICGA may reveal choriocapillary ischemia with hypofluorescence in the early stages^9^.

Although the imaging techniques mentioned above can help to diagnose most cases of CSC and differentiate from other diseases, FA and ICGA are invasive techniques that require a high demand for trained technicians and patient cooperation. Recently, OCT angiography (OCTA) has emerged as a new imaging technology. Dyeless OCTA is a fast, easy, and noninvasive technique, combining Doppler OCT and en face OCT^10,11^. More importantly, OCTA detects the movement of red blood cells, which facilitates blood flow imaging and provides the ability to analyze blood flow in different layers^10,12^. Because abnormal choroidal blood flow is considered to be important in the pathogenesis of CSC, OCTA may provide additional information about blood supply in CSC, and may help to better understand the underlying pathophysiology of the disease. Previous studies^7,13–16^ have described findings of OCTA with CSC. Hence, the aim of the present study was to describe OCTA findings in Asian patients with chronic and acute CSC, and to compare OCTA with traditional angiographic imaging modalities.

## Methods

### Patients

This study was performed at the Department of Ophthalmology, Peking University People’s Hospital, Beijing, China. All patients diagnosed with symptomatic CSC between February 2016 and January 2017 were included. The inclusion criteria were as follows: presence of active focal or diffuse leakage on FA; presence of SRF with or without serous PED on OCT; and presence of abnormal dilated choroidal vasculature on ICGA. Patients with other ocular conditions commonly associated with SRF, such as choroidal neovascularization, polypoidal choroidal vasculopathy (PCV), diabetic retinopathy, retinal vascular occlusion, Coat’s disease, or any disease that may affect the quality of imaging (quality of OCTA images <6), such as cataract, high myopia or nystagmus; history of ocular surgeries including retinal laser; pregnancy; any uncontrolled systemic disease; or any condition rendering patients intolerable to image acquisition were excluded. The research protocol adhered to the tenets of the Declaration of Helsinki. Informed consent was obtained from all subjects, and the protocol was approved by Ethics Committee of People’s Hospital of Peking University.

All patients underwent a comprehensive ophthalmological examination, including best-corrected visual acuity (BCVA), intraocular pressure, slit-lamp biomicroscopy, dilated fundus examination, FA (FF 450plus, Carl Zeiss Meditec AG, Germany), ICGA (Heidelberg Engineering, Heidelberg, Germany) and OCTA (CIRRUS HD-OCT Model 5000, Carl Zeiss Meditec, Germany). The spectral domain OCT instrument uses an algorithm, known as OCT microangiography-complex, that is capable of scanning at a rate of 68,000 A-scans per second and introduces improved tracking software known as FastTracTM retinal-tracking technology^17^. All studied eyes were scanned using Angio scan 6 × 6 mm section, 3 × 3 mm section, and HD 21 lines. The built-in OCTA software was used to manually alter the automated segmentation if necessary. All OCTA examinations were performed by one well-trained physician (HJ) on the same day when FA and ICGA were performed to maximize the comparability of the different modalities.

**Figure 1:**
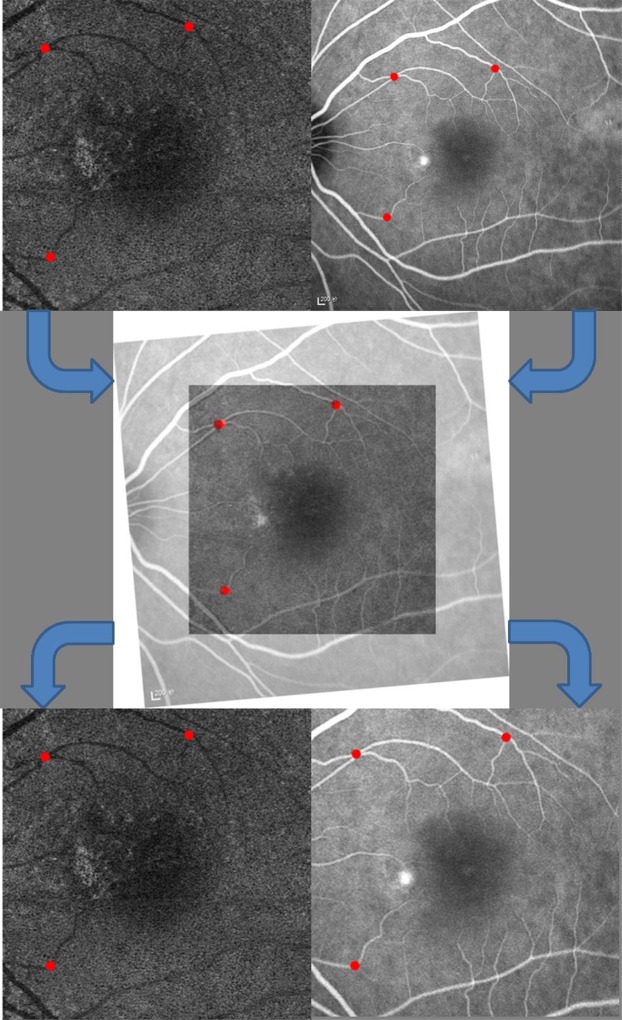
A representative example of image registration. Three user-specified landmarks on 6 × 6 mm en face choriocapillary optical coherence tomography angiography image and mid-phase indocyanine green angiography image were accurately matched by translating, rotating, and rescaling target images. After the registration, two same size (6 × 6 mm) images from the two modalities were obtained for further comparison and Jaccard index measurement.

Two experienced masked observers (QJF and ZMW) independently read the OCTA and ICGA images. En face choriocapillary OCTA images and representative ICGA images at early (within 30 s post-injection) and mid phase (60–180 s post-injection) were exported in JPG format and imported into ImageJ (version 1.4.3.67, 2016) for qualitative analysis. The OCTA images and ICGA images were aligned using Photoshop CS6 (version 13.0.0.0, Adobe, San Jose, CA, USA). After translation, rotation, and rescaling the target images, three user-specified landmarks on both modalities were accurately matched. A representative example of registered images is shown in Fig. 1.

Each observer annotated abnormal areas on en face choriocapillary OCTA images, hyperfluorescence area on mid-phase ICGA images, and hypofluorescence area on early-phase ICGA images. Adjacent abnormal areas that appeared confluent to each other were amalgamated into a single area.

To determine interobserver agreement of image annotations, spatial overlap of the annotations of type A on en face choriocapillary OCTA and the hyperfluorescence area on mid-phase ICGA was calculated separately according to Jaccard index (JI) as follows:

$${\rm{J}}({\rm{A}},{\rm{B}})=\frac{|{\rm{A}}{\cap }^{}{\rm{B}}|}{|{\rm{A}}{\cup }^{}{\rm{B}}|}=\frac{|{\rm{A}}{\cap }^{}{\rm{B}}|}{|{\rm{A}}|+|{\rm{B}}|-|{\rm{A}}{\cap }^{}{\rm{B}}|}$$

**Figure 2:**
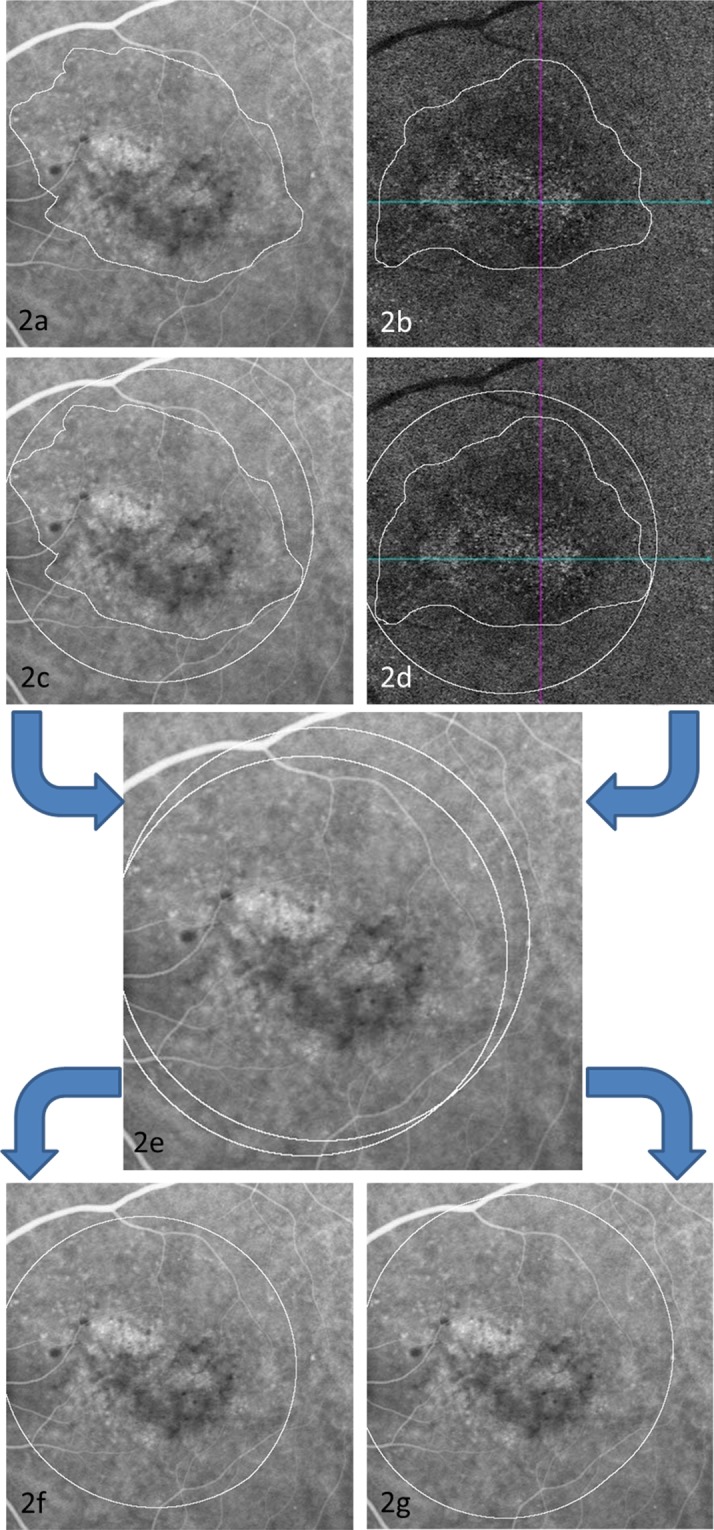
Jaccard index (JI) calculation. Two annotations of region-of-interest were performed on two images first (**a**,**b**); the circumcircle was annotated using Image J, respectively (**c**,**d**); the two images were overlapped using ImageJ (**e**); The annotation on (**f**) was the intersection of the two circumcircles; The annotation on 2 g was the union of the two circumcircles; the agreement of two annotations was measured according to JI, JI = 2 f/2 g.

A and B represent the area of the smallest rounds that covered the annotation of grader A and grader B. JI was the ratio of intersection area of the two rounds divided by the union area of the two rounds. A representative example of how they were defined is shown in Fig. 2. The spatial correspondence of type A on en face choriocapillaris OCTA and the hyperfluorescence area on mid-phase ICGA was measured in the same manner according to JI.

A qualitative analysis and comparison of the entire imaging data set were conducted. Statistical analysis was performed using a commercially available spreadsheet program (Excel version 14.0; Microsoft Corporation, Redmond, WA, USA). The Student’s t-test for paired samples and Chi-squared test were used for statistical analysis; P < 0.05 was considered to be statistically significant.

## Results

This study included 72 eyes of 64 patients (17 women and 47 men) with a mean age of 46.3 ± 10.5 years. Mean BCVA was 0.35 ± 0.29logMAR. 56 patients exhibited a unilateral involvement, whereas the disease occurred bilaterally in 8 patients. Of the 72 eyes, 44 (61.1%) were diagnosed with acute CSC and the remainder (38.9%) with chronic CSC.

We found 54 eyes (75%) presenting double-layer sign (DLS) in OCT B-scans. 6 of them (mean age of 57, 4 men and 2 women) were found abnormal vascular network, which could be choroidal neovascularization (CNV) or branching vascular network (BVN), within the DLS on OCTA images after manually adjusting the slab boundary just above and under the DLS. These 6 eyes were excluded for the following analyze of OCTA images and the comparison of OCTA and ICGA images. Patient demographics and clinical characteristics of the remained 66 eyes are listed in Table 1.

**Table 1:** Demographic information of patients with central serous chorioretinopathy BCVA, best-corrected visual acuity.

Characteristic	Value
Sex	
Male	43 (48 eyes)
Female	16 (18 eyes)
Age (years), mean ± SD (range)	45.4 ± 9.5 (23–65)
BCVA (logMAR)	0.32 ± 0.28 logMAR
Symptom duration	
Acute (≤6 months)	42 eyes
Chronic (>6 months)	24 eyes
Eye involvement, n	
Unilateral	52
Bilateral	7

**Figure 3:**
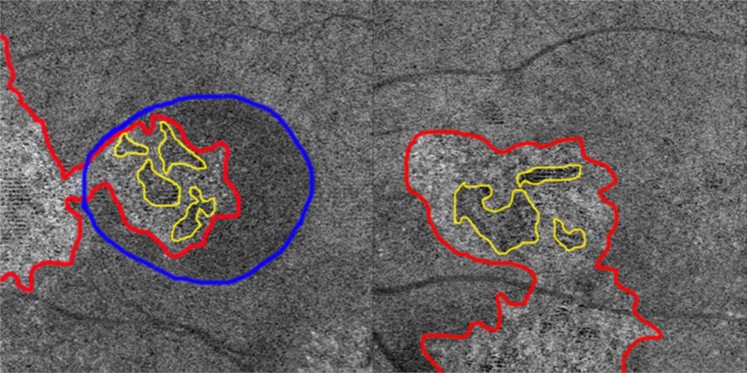
Three main types of abnormalities were found in choriocapillaris optical coherence tomography angiography (OCTA). Type A (outlined in red), was defined as coarse granulated high reflective area; type B (outlined in blue) was defined as the roundish dark halo around type A; and type C (outlined in yellow) was defined as the coarse granulated low reflective spot inside type A. The left panel image shows a representative case with all the three types of abnormalities on choriocapillaris OCTA. The right panel is a representative case without type B abnormality.

Abnormal choriocapillaris OCTA images were noticed in all 66 remained eyes. According to the abnormal patterns, three types of abnormalities were defined: type A, coarse granulated high reflective area (outlined in red in Fig. 3); type B, roundish dark halo around type A (outlined in blue in Fig. 3); and Type C, coarse granulated low reflective area (outlined in yellow in Fig. 3). Type A, B and C abnormalities were present in 61 (92.4%), 54 (81.8%), 66 (100%) eyes, respectively. There were 54 (81.8%) eyes that exhibited all three types abnormalities, 7 (10.6%) had type A and C abnormalities, and 5 (7.6%) only had type C abnormalities.

**Figure 4:**
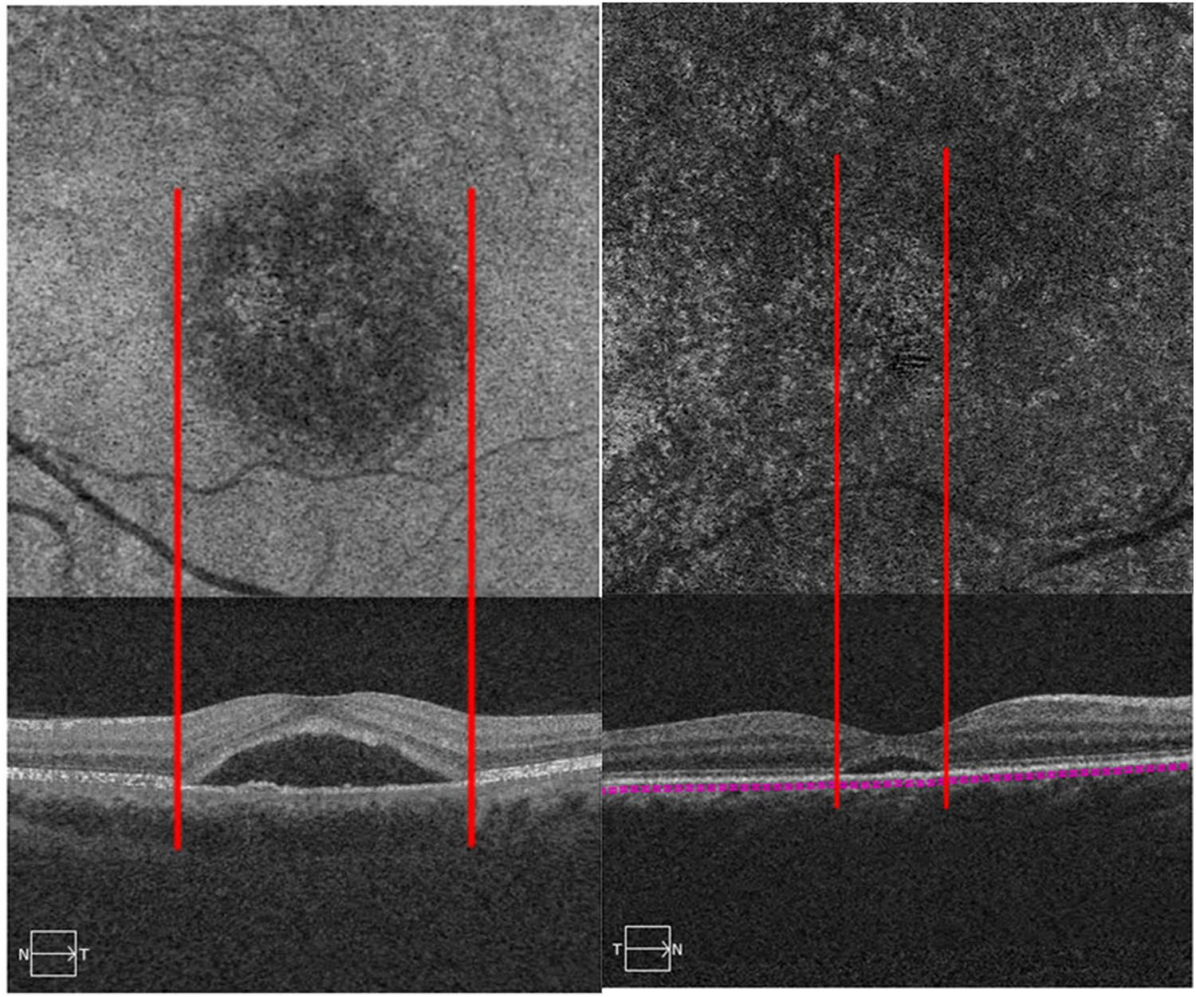
En face choriocapillaris optical coherence tomography angiography (OCTA) images and the B-scan of the same central serous chorioretinopathy (CSC) patients. Patient with type B (left panel) showed the extent of subretinal fluid (SRF) on B-scan corresponding with the area of type B abnormalities on OCTA. Patients with a thinner SRF did not exhibit the corresponding halo on OCTA (right panel).

All type B abnormality collocated with SRF on structural OCT. Patients with type B abnormality exhibited a greater mean maximum SRF thickness than patients without type B abnormalities (538.0 ± 230.8 µm versus 242.8 ± 180.1 µm; P < 0.01) (Fig. 4). Type B abnormality presented in 36 eyes with acute CSC and 13 eyes with chronic CSC (54.5% vs. 19.7%, respectively; P = 0.001).

**Figure 5:**
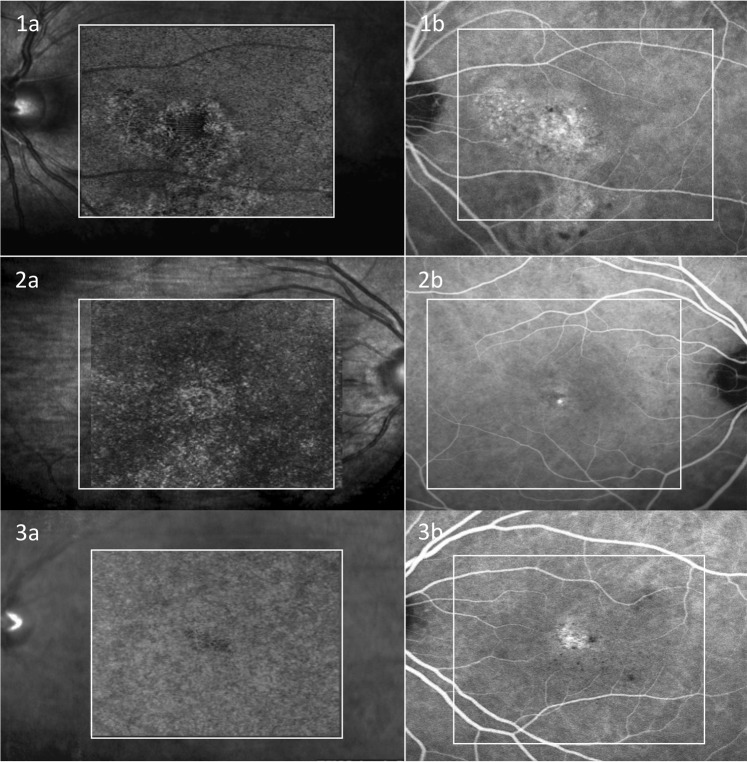
Comparison of type A abnormality on En face choriocapillaris OCTA and the hyperfluorescence area on ICGA in representative patients. The left column were OCTA images overlaid to the infra-red fundus images and the right column were ICGA images. The images in the white square were the OCTA scanned area after registration with ICGA images. Case 1 showed the type A abnormality on OCTA (**1a**) was equal to the hyperfluorescence area on ICGA (**1b**). Case 2 showed the type A abnormality on OCTA (**2a**) was larger than the hyperfluorescence area on ICGA (**2b**). Case 3 showed there was only type C but no type A abnormality on OCTA (**3a**), while there was prominent hyperfluorescence area on ICGA (**3b**).

Type A abnormalities presented in 61 eyes with CSC. The mean JI, which indicate the interobserver agreement of type A on OCTA and the hyperfluorescence area on ICGA, was 0.92 ± 0.05 and 0.93 ± 0.04 respectively. The mean JI of type A on OCTA and the hyperfluorescence area on ICGA was 0.84 ± 0.15 for grader 1 and 0.82 ± 0.23 for grader 2. The area of type A abnormality on OCTA and the hyperfluorescence area on ICGA were decided after adjudication between the two graders and then compared with one another in the same patient. If the difference was within 10% of the hyperfluorescence area on ICGA, they were defined as equal. The area of type A abnormality on OCTA was equal to the hyperfluorescence area on ICGA in 49 (74.2%) eyes and larger than the hyperfluorescence area on ICGA in 12 (18.2%) eyes (Fig. 5). Type A area on OCTA had a statistically larger area than hyperfluorescence on ICGA (12.7 ± 3.8 mm^2^ vs 11.9 ± 4.4 mm^2^; P = 0.01 [paired t-test]). The mean area of type A abnormalities on OCTA was 12.8 ± 4.0 mm^2^ in acute CSC patients and 13.4 ± 3.7 mm^2^ in chronic CSC (Table 2). There is no significant difference of type A area between acute and chronic patients (P = 0.72).

**Table 2:** Comparison of type A abnormality area on OCTA and hyperfluorescent area on ICGA.

Item	Area		Mean Jaccard index
	Grader 1	Grader 2	
Hyperfluorescence on ICGA (mm^2^)	11.9 ± 4.4	12.0 ± 4.5	0.93 ± 0.04
Type A on OCTA (mm^2^)	12.7 ± 3.8	12.8 ± 3.9	0.92 ± 0.05
Mean Jaccard index	0.84 ± 0.15	0.82 ± 0.23	—

OCTA, optical coherence tomography angiography; ICGA, indocyanine green angiography.

**Figure 6:**
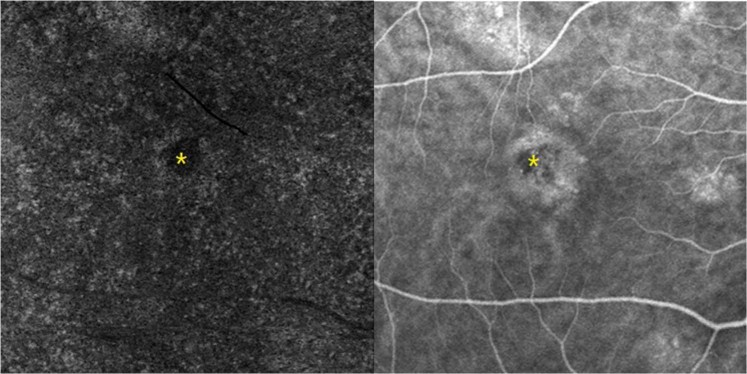
En face choriocapillary optical coherence tomography angiography (OCTA) with type C (yellow asterisk on left panel) and contemporary early-phase indocyanine green angiography (ICGA) with hypofluorescent area (yellow asterisk on right panel) in a 31-year-old man.

Type C abnormalities were observed in all 66 eyes. Type C area corresponded with the hypofluorescent area on early-phase ICGA; however, the former was larger than the latter in all eyes with the mean area of 0.22 ± 0.14 mm^2^ and 0.10 ± 0.07 mm^2^, respectively (Fig. 6).

## Discussion

Although CSC is an ancient disease, its pathogenesis remains unclear. It is assumed that high levels of corticosteroid and catecholamine, coagulation and circulating antigen-antibody complexes in vessels, and compression of pachychoroid vascular to choriocapillaris, results in dysfunction and ischemia of the choroidal capillaries, compensatory hyperpermeability and leakage of the around choroidal capillaries and, subsequently, cause dysfunction in RPE, which leads to accumulation of fluid under the neuroretina. OCTA is a newly developed ophthalmic imaging technology, which enables us to view different layers of blood flow and perfusion in the retina and choroid. OCTA may facilitate the elucidation of the pathogenesis of CSC.

Recent studies have focused on multimodal imaging assessment of CSC^7,9,14,16,18–21^; however, only a few have performed quantitative analysis, and different OCTA findings in CSC have been reported among investigators. Teussink^13^ *et al*. compared OCTA and ICGA in 18 eyes with chronic CSC. They found reduced flow surrounded by hyper-perfused areas on choriocapillaris OCTA. Chan *et al*.^20^ studied 12 eyes with acute CSC and 14 with chronic CSC, and found high signal intensity on choriocapillary OCTA in all eyes and believed this pattern demonstrated dilated capillaries of the choroid.

In our study, we found 3 types of abnormalities at the level of choriocapillaris on OCTA. The most common abnormality was the coarse granulated low reflective spot (Type C), which was found in all eyes. Since we have known that various factors such as PED, and subretinal fibrin might cause shadow artifacts16, We had used combinations of OCT B scan and color fundus photograph to exclude shadow artifact at choriocapillaris level caused by PED, fibrin and also the migration of pigment. The low signal area caused by shadow effect was not included in our type C area in our study. Shinojima *et al*. had also found low-flow signal in all their 58 studied CSC eyes. They thought it might indicate the thinned choriocapillaries being pushed upward by pachyvessles or low blood flow at the choriocapillaries level16. Our finding demonstrates the reduced choroidal blood flow in CSC and is consistent with other earlier reports. Scheider *et al*. found delayed choroidal filling on ICGA at the site of the hot spot in 82% of CSC patients^22^. Kitaya *et al*. had found delays in arterial filling in 75% CSC eyes on ICGA and the choroidal blood flow in CSC eyes was 45% lower than in fellow eyes^23^. Comparison of OCTA and ICGA images revealed that the type C abnormality area corresponded with the hypo-fluorescence area on early-phase ICGA; however, the former was larger than the latter in all eyes. This might because the hypo-fluorescence area on early-phase could be easily blocked by the leakage of choriocapillary or stain from subretinal fibrin content in later phase ICGA. And these finding suggest that OCTA might be more valuable than ICGA in revealing choriocapillary hypo-perfusion.

In our study, the type A abnormality, which was the coarse granulated high reflective area, was present in 61 (92.4%) eyes. In the study by Chan *et al*., however, high signal intensity on choriocapillary OCTA was observed in all eyes; the different result may be explained by the smaller sample size of their study^20^. Although it remains unclear what the type A represents, since there was high spatial correspondence between type A abnormality on OCTA and hyperfluorescence area on ICGA (mean JI 0.84 ± 0.15 for grader 1 and 0.82 ± 0.23 for grader 2), we presume it was caused by the dilation and increased blood flow of choriocapillaries in CSC patients. RPE atrophy may also contribute to the type A abnormality in chronic CSC eyes, but by comparing the OCTA images with the FA and OCT B scan images, type A area was much larger than the RPE atrophy area in certain eyes. Furthermore, if type A area was merely artifacts caused by the atrophy of RPE due to long lasting SRF, it should be more frequent in the chronic CSC than the acute CSC since RPE atrophy was more frequent in the chronic CSC, but in fact we found there is no significant difference of type A area between acute and chronic patients (P = 0.72). Automatic segmentation error might also cause artifacts and affect the abnormal finding at choriocapillaris level, one must be cautious in the interpretation of all types of abnormalities at the level of choriocapillaris on OCTA.

The mean type A area was larger than high permeability area on ICGA (12.7 ± 3.8 mm^2^ vs 11.9 ± 4.4 mm^2^; P = 0.01[paired t-test]) in our study. 49 eyes (74.2%) exhibited an equal area of type A abnormality on OCTA and hyperfluorescence area on ICGA, which was consistent with the findings in the study by Teussink *et al*.^13^, while 12 eyes exhibited a larger highly reflective area on OCTA than on ICGA, and 5 eyes did not present type A abnormality on OCTA at all. This finding may be explained by the different mechanism of OCTA and ICGA imaging. OCTA depicts blood flow dependent on intravascular cell movement and maybe affected by the velocity of blood flow. If the blood flow is too high or too slow, it may be beyond the test limitations of the OCTA device, and we would not be able to detect the abnormality. Although ICGA depicts blood flow independent of intravascular cell movement, as long as there is plasma, it is visible on ICGA. Different imaging modalities provide blood flow information that is supplementary to one another. Another possible explanation is that the dilation of choriocapillaries and increased blood flow within them may not present in all CSC patients, or not in all stages of CSC.

In our study, the type B abnormality exhibited a very regular round or oval shape, and presented in 36 eyes with acute CSC and 13 eyes with chronic CSC (87.8% vs. 52%; P = 0.001). It was caused by the shadow effect of SRF because we found it co-located with SRF on structural OCT in all eyes. Patients with the type B abnormality had a greater mean maximum SRF thickness than patients without type B abnormalities. We postulate the thickness and the composition of the SRF may be an influential factor in the shadow effect.

The inter-observer agreement for type A on OCTA and hyperfluorescence area on ICGA was higher than Teussink *et al*. reported^13^. That was mainly because we were using a circumcircle of the abnormal area instead of the border of the abnormal area on OCTA to calculate the JIs. The reason why we chose to use different methods was as follows. First, the border between the appearance of healthy and affected area on OCTA may not easily be drawn between graders, even though the appearance of these changes was significantly different from the fairly homogeneous appearance of the healthy choriocapillary layer. Second, we used a round laser spot that covered the choriodal lesion related to the active leakage when applying PDT to CSC patients, we believe a circumcircle would be more practical in our clinic. Our study also revealed a higher agreement of type A abnormality on OCTA and hyperfluorescence on ICGA, and this result may provide the opportunity to use OCTA to guide PDT for active CSC patients. Whether OCTA-guided PDT is equally as effective as ICGA-guided PDT still needs further prospective study to draw a definitive conclusion.

Among OCTA studies of CSC, our investigation included the most number of patients; nevertheless, it was still limited by cross-sectional data acquisition. We included treatment-naïve patients only and excluded potential confounders of different interventions on OCTA images. However, the manual segmentation and annotation methods could also be a factor that may have introduced bias in the result. Because of the limited length of each OCTA scan, we only compared the foveal-centered OCTA images with the ICGA images at the corresponding position and did not perform the OCTA image montage because it was time prohibitive. We were using spectral domain OCTA but not swept source OCTA instrument in this study and it is not satisfying for choroidal circulation at this moment because of OCT light attenuation due to RPE. Because of this limitation we only analyzed the choriocapillary layer but not the Sattler and Haller layer. Nevertheless, we believe that with development of OCTA technology, wide-field swept source OCTA images would provide more information.

In conclusion, OCTA enables noninvasive visualization of the retinal and choroidal vasculature in CSC patients. Abnormalities were found in all en face choriocapillaris OCTA images of CSC. The most common abnormality was the coarse granulated low reflective area and the coarse granulated high reflective area on en face choriocapillaris OCTA, which suggested choriocapillary ischemia and compensatory dilation were important in the pathogenesis of CSC. Coarse granulated high reflective area in OCTA corresponded well with the hyper-permeability area in ICGA and may guide PDT instead of ICGA in the future.
